# Midlife stress-related exhaustion and dementia incidence: a longitudinal study over 50 years in women

**DOI:** 10.1186/s12888-024-05868-z

**Published:** 2024-07-11

**Authors:** Xinxin Guo, Tore Hällström, Lena Johansson, Jenna Najar, Hanna Wetterberg, Simona Sacuiu, Silke Kern, Ingmar Skoog

**Affiliations:** 1 Neuropsychiatric Epidemiology, Section of Psychiatry and Neurochemistry, Institute of Neuroscience and Physiology, Sahlgrenska Academy, University of Gothenburg, Gothenburg, Sweden; 2 Department of psychiatry, Affective Disorders, Sahlgrenska University Hospital, Gothenburg, Sweden; 3 Institute of Health and Care Sciences, Sahlgrenska Academy, University of Gothenburg, Gothenburg, Sweden; 4 Department of Addiction and Dependency, Sahlgrenska University Hospital, Gothenburg, Sweden; 5 Department of neuropsychiatry, Sahlgrenska University Hospital, Gothenburg, Sweden; 6 Section Genomics of Neurodegenerative Diseases and Aging, Department of Human Genetics Amsterdam UMC, Vrije Universiteit Amsterdam, Amsterdam UMC, Amsterdam, The Netherlands; 7 Infection medicine, Department of Clinical Sciences Lund, Lund University, Lund, Sweden

**Keywords:** Stress, Exhaustion, Dementia, Longitudinal study, Women

## Abstract

**Backgrounds:**

Cognitive problems are common symptoms among individuals with stress-related exhaustion. It is still unknown whether these individuals are at a higher risk of developing dementia later. This study aims to examine the relationship between midlife stress-related exhaustion and dementia incidence.

**Methods:**

A population sample of 777 women (aged 38, 46, 50 and 54 years) without dementia at baseline was followed over 50 years, from 1968 to 2019. Stress-related exhaustion was based on information from the psychiatric examination in 1968/69. Information on dementia incidence between 1968 and 2019 was obtained from neuropsychiatric examinations, key-informant interviews, and hospital registry. Dementia was diagnosed according to the DSM-III-R criteria. A subgroup of non-demented women (*n* = 284) was examined for cognitive functions by the Gottfries-Bråne-Steen scale 24 years after baseline.

**Results:**

Stress-related exhaustion in midlife was associated with higher risk for development of dementia before age 75 (Hazard ratio and 95% confidence interval: 2.95 and 1.35–6.44). The association remained after adjustment for age, major depression, and anxiety disorder. Mean age of dementia onset was younger for women with stress-related exhaustion than women without stress (mean ± SD, 76 ± 9 vs. 82 ± 8 . *p* = 0.009). Women with stress-related exhaustion in midlife still showed more cognitive impairments 24 years later compared with women without stress (Odds ratio and 95% confidence interval: 2.64 and 1.15–6.06).

**Conclusions:**

We found that women with stress-related exhaustion in midlife were at a higher risk to develop dementia at relatively younger age. These women showed persistently lower cognitive functions over years even without dementia. Present study results need to be interpreted with caution due to small sample size and should be confirmed in future studies with larger sample size. Our study findings may imply the importance of long-term follow-up regarding cognitive function among individuals with stress-related exhaustion.

## Background

Increasing evidence suggests that severe stress is an important risk factor of cognitive decline [1–8]. Our earlier studies found that women who experienced chronic stress in midlife had higher risk for development of Alzheimer’s disease (AD) [1]. Stress in midlife was associated with temporal lobe atrophy and small vessel disease on brain MRI in later life [9]. Moreover, women with midlife stress showed higher levels of neurodegenerative biomarkers in cerebrospinal fluid (CSF) measured in older age, including total tau protein, amyloid beta 40, visinin-like proteion-1 and myelin basic protein [10, 11]. In line with our studies, large population studies in Finland (CAIDE) demonstrated that midlife work-related stress increased the risk for mild cognitive impairment, dementia, and AD [3, 4]. Higher levels of midlife work-related stress were associated with poorer performance on global cognition, slower processing speed, as well as grey matter atrophy on brain MRI [12]. Three population studies also reported that the risks of dementia increased among individuals with stress-related psychiatric disorders registered in midlife compared with individuals without stress-related disorders [5–7]. These results suggest that longstanding exposure to psychological distress may lead to severe and unreversible brain damages.

Individuals react differently to the same stressor, which may be affected by various factors, such as different coping strategies, cognitive perception, environment during childhood, genetic factors, and personality [13, 14]. Some endure stress without development of symptoms, whereas others react with severe symptoms, for example, depression, anxiety, and exhaustion. In 2003, The Swedish National Board of Health and Welfare proposed diagnostic criteria for exhaustion disorder [15], and diagnostic code F43.8 A was assigned in the Swedish ICD-10. Exhaustion disorder is characterized by physical and mental exhaustion as a result of stressors (both at and outside work) for at least 6 months, and symptoms should cause clinically significant distress and impairment in social, occupational, or other important areas of functioning. Exhaustion disorder is different from psychological distress, anxiety and depression. Psychological distress is a mental health outcome typified by psychophysiological and behavioural symptoms that are not specific to a given mental pathology [16, 17]. It includes one or several symptoms of anxiety, depression, irritability, declining intellectual capacity, tiredness, sleepiness, and so forth [16, 17]. Individuals suffered from psychological distress vary in functional level and most of them don’t have severe functional impairments that require medical care and intervention. Depression (loss of interest, unhappiness, and hopelessness), and anxiety (restlessness, feeling tens) are common comorbidities in individuals with exhaustion disorder. However the main symptom of exhaustion disorder is extreme tiredness causing significant functional impairment, and tiredness is often more severe and long-lasting than depression and anxiety [18]. Cognitive problems of memory and concentration are other prominent symptoms in people with exhaustion disorder [19, 20] and often persist many years [21]. However, it is still unknown whether people with stress-related exhaustion are at higher risks of dementia, as longitudinal studies are rare.

## Methods

### Aim and study design

Based on a female population followed for 50 years, this study aims to examine the relation between midlife stress-related exhaustion and dementia incidence. We will also compare risks of incident dementia between two groups of women reporting longstanding stress in midlife, one group with symptoms of exhaustion and the other without symptoms of exhaustion.

### Study population

The study is part of the Prospective Population Study of Women in Gothenburg [22], which was initiated in 1968/69 (baseline). The study population was systematically sampled from the Swedish Population Register based on specific birth dates to yield a representative sample at the ages studied. Altogether 800 women at age of 38, 46, 50 and 54 years participated in the psychiatric examination in 1968/69 (participation rate 89%). Complete data were obtained from 777 women (38-year-olds *n* = 105, 46-year-olds *n* = 301, 50-year-olds *n* = 284, 54-year-olds *n* = 87). Mean age at baseline was 47 years. None of the women had dementia at baseline. Follow-ups were performed in 1974/75, 1980/81, 1992/93, 2000/01, 2005/06, 2009/11, 2015/16, and 2018/19 [23–25].

### Assessment of chronic stress and stress-related exhaustion

Assessment of stress-related exhaustion was based on information from the psychiatric examination at baseline 1968/69. The examination was performed by an experienced psychiatrist, one of the coauthors (TH). The examination was semi-structured, allowing for clarifying questions, and included a comprehensive psychiatric interview and observations of mental symptoms during the interview [26]. Current psychiatric symptoms and signs as well as symptom duration were rated, such as tiredness, concentration problems, memory problems, tearfulness, hypersensitivity, irritability, loss of ambition, phobia, anxiety, vegetative symptoms, feeling of tension, sleep disturbance, and depressive symptoms. A global assessment of function and degree of disability was made by the examinator after the interview.

Presence of chronic stress was rated according to a standardized question: “Have you experienced any period of stress (1 month or longer) in relation to circumstances in everyday life, such as work, health or family situation? Stress refers to feelings of irritability, tension, nervousness, fear, anxiety, or sleep disturbances.” Participants were asked to choose between; 0 = have never experienced any period of stress during the last 5 years; 1 = have experienced period/s of distress more than 5 years ago; 2 = have experienced one period of distress during the last 5 years; 3 = have experienced several periods of distress during the last 5 years; 4 = have experienced constant distress during the last year, or 5 = have experienced constant distress during the last 5 years. In the current study, chronic stress was defined as a rating of 3–5.

Women were considered to have stress-related exhaustion if all the following criteria were met. (A) Experience of chronic stress. (B) Presence of physical and mental exhaustion. (C) Problems of concentration and/or memory. (D) Presence of at least 2 other symptoms, including hypersensitivity to sensory stimuli, emotional lability or irritation, sleep problems, and stress-related physical symptoms. (E) Duration of the disturbance (criteria B, C, D) is more than 3 months. (F) The disturbance is accompanied by significant distress or impairment in cognitive, social, occupational, or other important areas of functioning. (G) The disturbance is not attributable to other psychiatric disorders (bipolar disorder, substance use disorder, schizophrenia, and other psychotic disorders). These criteria of stress-related exhaustion are compatible with the diagnose criteria of exhaustion disorder by Swedish National Board of Health and Welfare [15]. If women met criterion A, but not all of the others, they were considered as stress without exhaustion.

### Diagnosis of dementia

Diagnosis of dementia was based on combined information from neuropsychiatric examination, close informant interview, and hospital register [25, 27]. *Neuropsychiatric examinations* were performed by experienced psychiatrists in 1974/75, 1980/81 and 1992/93, and by experienced psychiatric research nurses in 2000/01, 2005/06, 2009/11, 2015/16, and 2018/19. All examinations were semi-structured. The examinations included ratings of common signs and symptoms of dementia, e.g. assessments of memory, orientation, general knowledge, apraxia, visuospatial function, understanding proverbs, following commands, naming ability, and language. Identical instruments were used since 1992, including the Comprehensive Psychopathological Rating Scale [28], Gottfries-Bråne-Steen (GBS)-scale, the Mini Mental State Examination [29], the Alzheimer’s Disease Assessment Scale [30] and the Clinical Dementia Rating [31]. *Close informant interviews* were performed by psychiatric nurses since 1992/93. The interviews were semi-structured and comprised questions about changes in behaviour and intellectual function, psychiatric symptoms, activities of daily living, and, when relevant, age at onset for stroke and dementia, and disease course. *The Swedish Hospital Discharge Register* provided information on diagnoses of all individuals discharged from hospitals on a nationwide basis since 1978. Diagnoses were classified according to the International Statistical Classification of Diseases and Related Health Problems (ICD).

Dementia was diagnosed according to the Diagnostic and Statistical Manual of Mental Disorders (DSM-III-R) criteria, as described previously [25, 27]. Dementia diagnoses were made by psychiatrists after reviewing information from both neuropsychiatric examinations and the close informant interview. The diagnosis was made if the participant had dementia according to both sources of information, or if there was clear evidence of dementia from one source and subthreshold symptoms from the other. For individuals lost to follow-up, dementia diagnoses were based on information from the Hospital Discharge Register. Age of dementia onset was based on the combined information from neuropsychiatric examination, close informants and register data.

### Assessment of cognitive function by GBS-scale

Cognitive function was examined by GBS scale [32] in the follow-up examination in 1992/93. A subgroup of women born in 1914 (78-year-olds, *n* = 29), 1918 (74-year-olds, *n* = 113), and 1922 (70-year-olds, *n* = 154) were examined. Twelve women developed dementia before 1992, leaving 284 for this study. The GBS scale is a comprehensive global assessment tool for evaluating dementia symptoms and is based on a semi-structured interview and observation of the patient. In this study, we used only the subscale for cognitive functions measuring orientation (to person, time, and place), short-term memory, long-term memory, wakefulness, concentration, ability to increase tempo, absentmindedness, longwindedness, and distractibility. Impairment of cognitive functions was estimated by research nurses or psychiatrist who performed the neuropsychiatric examination. As only few women presented impairment with each of the cognitive functions, the number of impaired cognitive function was used in the analyses.

### Assessments of characteristics at baseline (in 1968/69)

Characteristics were based on self-reported information. Education was dichotomized as 6 years and more, or less than 6 years. Family income was defined as yearly total family income divided by number of family members living at home, and the lowest decentile was defined as low family income. Marital status was classified as married or unmarried. Working status was defined employment (part-time or fulltime) and unemployment. Hypertension was defined as systolic blood pressure ≥ 140 mm Hg and/ or diastolic blood pressure ≥ 90 mm Hg and/or taking antihypertensive medication. Diagnosis of major depression was performed based on the psychiatric examination at baseline, and was revised later according to DSM-III [33]. Cases of anxiety disorder were identified from the dataset 1968/69 using algorithms based on the DSM-IV criteria. Anxiety disorder included social phobia, panic disorder, agoraphobia, generalized anxiety disorder, and obsessive-compulsive disorder. History of heart infarct, stroke, diabetes, and chronic bronchitis was reported by participants.

### Statistical methods

Cox regression was used to examine the association between stress with/without exhaustion at baseline and total incident dementia. As there is a significant age interaction for the association between stress-related exhaustion and dementia incidence, we examined the association by stratifying dementia onset before age 75, between age 75 and 85, and after age 85. Women reporting no stress in the last five years before baseline were regarded as the reference group. Hazard ratio (HR) and 95% confidence interval (95% CI) were adjusted by cohort, age, education, low family income, hypertension, major depression and anxiety disorder at baseline. Two sampled t-test was performed to study the difference of age at dementia onset between women with stress-related exhaustion and women reporting no stress. Competing risk for death was calculated by the Stata procedure STCRREG. We also examined the effect of stress with/without exhaustion on risk of dementia before and after age 75 by propensity matching scores. Propensity matching scores were calculated by the Stata procedure TEFFECTS PSMATCH.

The associations between chronic stress with/without exhaustion at baseline and number of impaired cognitive functions (0/1/>1) examined by GBS was tested by ordinal logistic regression. Women reporting no chronic stress in the last five years before examination 1968/69 were regarded as the reference group. Odds ratio (OR) and 95% CI are presented after adjustment by age and education. *P* < 0.05 is considered as statistically significant.

## Results

Among 777 women examined at baseline, 58 women (7.5%) had stress-related exhaustion and 80 (10.3%) had stress but not exhaustion. Characteristics of participants are presented in Table 1. Compared with women who did not experience stress, women with stress-related exhaustion were older, worked less, and were more likely to have major depression, anxiety disorder, and chronic bronchitis. These two groups were similar regarding education, number of children, marital status, economic status and hypertension. Compared with women experiencing stress but not exhaustion, women with stress-related exhaustion had higher prevalence of major depression (14% vs. 41%) and anxiety disorder (30% vs. 67%), and reported more frequently constant stress in the last 5 years (11% vs. 31%). No difference regarding other baseline characteristics between these two groups.

**Table 1:** Characteristics of participants at baseline (*n* = 777)

	Stress with exhaustion (*n* = 58)	Stress without exhaustion (*n* = 80)	No stress (*n* = 639)
Age			
38-years-old 46-years-old 50-years-old 54-years-old	8.6% 41.3% 20.7% 29.3%*	6.3% 37.5% 45.0% 11.3%	14.9% 38.7% 36.9% 9.5%
Education > 6 years	28.7%	24.1%	23.9%
Employment	46.6%*	57.5%*	70.1%
Number of children 0 1–2 > 2	12.1% 70.7% 17.2%	21.3% 50.0% 28.7%	17.2% 54.3% 28.5%
Married	70.7%	71.3%	82.2%
Low family income	12.5%	14.3%	10.0%
Major depression	41.4%*	13.8%*	3.8%
Anxiety disorder	66.7%*	29.7%*	13.5%
Heart infarct	0	0	0
Stroke	0	0	0
Diabetes	0	0	0.6%
Chronic bronchitis	8.6%*	1.3%	1.6%
Hypertension	41.4%	32.9%	40.8%

*p < 0.05. Reference group is women who did not experience longstanding psychological stress in the last 5 years before baseline

### Dementia incidence 1968–2019

Among 777 women, 227 (29.2%) women developed dementia during 26,111 person-years. Total cumulative dementia incidence during the whole follow-up time was 31.0% for women with stress-related exhaustion, 33.8% for women reporting stress but not exhaustion, and 28.5% for reference group (women not experiencing chronic stress in the last 5 years). Dementia incidence before age 75 was 13.4%, 8.8% and 5.0%, respectively, for these three groups. Compared with the reference group, age at dementia onset was younger for women with stress-related exhaustion (mean ± SD, 82 ± 8 vs. 76 ± 9, *p* = 0.009), but not for women reporting stress but not exhaustion (82 ± 8 vs. 81 ± 9, *p* = 0.519).

Stress-related exhaustion was associated with higher risk for development of dementia before age 75, but not after age 75 (Table 2). This association before age 75 remained after adjustment for anxiety disorder, major depression, and other confounders at examination 1968/69. However, stress without exhaustion was not statistically significantly associated with dementia incidence before or after age 75 (Table 2). We also performed sensitivity analysis accounting competing risk of death. It didn’t change the associations between stress-related exhaustion and dementia (before age 75, HR (95% CI): 2.99 (1.35–6.62); after age 75, HR (95% CI): 0.97 (0.50–1.90)), neither did the associations between stress without exhaustion and dementia (before age 75, HR (95% CI):1.78 (0.78–4.07); after age 75, HR (95% CI):1.14 (0.73–1.79)). Propensity matching scores gave similar results as the Cox regressions.

**Table 2:** Relationships between midlife stress with/without exhaustion and incident dementia from 1968 to 2019 in women

	Stress with exhaustion (*n* = 58)	Stress without exhaustion (*n* = 80)
**Total dementia (** ***n*** ** = 227)**		
HR1 (95% CI) HR2 (95% CI) HR3 (95% CI)	1.37 (0.84–2.23) 1.45 (0.89–2.37) 1.35 (0.76–2.38)	1.24 (0.82–1.85) 1.20 (0.79–1.83) 1.17 (0.76–1.78)
**Dementia onset before age 75 (** ***n*** ** = 47)**		
HR1 (95% CI) HR2 (95% CI) HR3 (95% CI)	2.95 (1.35–6.44)* 3.01 (1.38–6.60)* 2.85 (1.11–7.28)*	1.89 (0.83–4.31) 1.94 (0.85–4.44) 1.64 (0.67-4.00)
**Dementia onset between age 75 and 85** (***n* = 94)**		
HR1 (95% CI) HR2 (95% CI) HR3 (95% CI)	1.06 (0.46–2.45) 1.13 (0.49–2.61) 1.09 (0.42–2.80)	1.31 (0.71–2.42) 1.28 (0.68–2.42) 1.36 (0.73–2.52)
**Dementia onset after age 85 (** ***n*** ** = 86)**		
HR1 (95% CI) HR2 (95% CI) HR3 (95% CI)	0.86 (0.32–2.38) 1.00 (0.36–2.77) 0.77 (0.23–2.52)	0.90 (0.43–1.87) 0.82 (0.38–1.80) 0.78 (0.36–1.72)

*P < 0.05. Reference group is women who did not experience longstanding psychological stress in the last 5 years before baseline

HR1 is adjusted by cohort and age

HR2 is adjusted by cohort, age, education, low family income and hypertension

HR3 is adjusted by cohort, age, major depression, and anxiety disorder

### Cognitive function 24 years after baseline among the non-demented women

Compared with the controls, women with stress-related exhaustion had higher number of impaired cognitive functions by the GBS-scale in examination 1992/93 (Table 3). However, women reporting stress but not exhaustion did not show more cognitive impairments than the controls.

**Table 3:** Relationships between stress with/without exhaustion at baseline and number of impaired cognitive functions by GBS in 1992/93 in the non-demented women

	Stress with exhaustion (*n* = 19)	Stress without exhaustion (*n* = 35)	No stress (*n* = 230)
Number of impaired cognitive functions			
0 1 > 1	21% (*n* = 4) 21% (*n* = 4) 58% (*n* = 11)	57% (*n* = 20) 9% (*n* = 3) 34% (*n* = 12)	48% (*n* = 111) 16% (*n* = 37) 36% (*n* = 82)
OR (95% CI)	2.64 (1.15–6.06)*	0.80 (0.40–1.60)	1.00 (reference)

* *p* < 0.05. OR is adjusted by age and education, tested by ordinal logistic regression. Reference group is women who did not experience longstanding psychological stress in the last 5 years before baseline

## Discussion

We followed a general women population over half a century and found that women who developed exhaustion syndrome after exposure to chronic stress in midlife were more likely to develop dementia before age 75 and developed dementia earlier than other women. Moreover, they still presented more cognitive impairments 24 years later even without dementia. Women reporting chronic stress but not exhaustion in midlife did not present more cognitive impairment or significantly higher risk of dementia incidence than those without chronic stress at baseline.

There are several possible explanations for the relationship between stress-related exhaustion and dementia. *First*, chronic stress may elevate stress hormone glucocorticoids, which may cause damage in hippocampus, a brain structure which is damaged early in Alzheimer’s disease and is well-known for its role in learning and memory. Hippocampus is particularly vulnerable to the neurotoxic effects of glucocorticoids because of its high density of glucocorticoid receptors [34]. In animal studies, chronic exposure to elevated levels of glucocorticoids has been linked to hippocampal neuronal loss, dendritic atrophy, reduced hippocampal volume [35–37] and decreased neurogenesis in the dentate gyrus region of the hippocampus [38]. In addition, exposure to chronic stress in both childhood and in adult life was associated with smaller volume in hippocampus and orbitofrontal cortex [9, 39–41]. Furthermore, chronic stress has been shown to trigger amyloid precursor protein misprocessing as well as increased plaque pathology, increased Aβ levels and hyperphosphorylated tau in animal models [42–44]. *Second*, stress may increase risk of dementia through other mechanisms, such as vascular damage and inflammation. Vascular damage is a well-established pathogenetic factor in both vascular dementia and AD [45, 46]. Many studies have reported association between stress and development of myocardial infarction, stroke, hypertension, and central redistribution of adiposity [47–53]. Chronic stress has been associated with elevated C-reactive protein, inflammatory cytokines, and arterial inflammation [48, 54]. Brain inflammation may give rise to both amyloid plaques and neurofibrillary tangles characteristic of AD [46, 55]. *Third*, it is well-known that AD pathologies often develop over years with start in middle age or even earlier [56]. For example, beta-amyloid fibrils and plaques present more than two decades before AD symptom onset [56]. Pathological brain changes may increase risk of both exhaustion and dementia.

We found that stress with exhaustion, but not stress without exhaustion, was significantly associated with cognitive impairment and also incident dementia before age 75. Explanations can be that women with stress-related exhaustion were exposed to more severe or longer stress, or were more vulnerable to stress than women without exhaustion. Individuals with vulnerabilities, such as low cognitive capacity, negative environment during growing up, personality disorders, high neuroticism, lack of coping strategies and health issues, may be more sensitive to stress and therefore likely to develop stronger and longer stress reactions even with common stressors, resulting in brain damage. The lack of statistically significant association between stress without exhaustion and dementia before age 75 may also be due to low sample size.

Stress-related exhaustion was related to development of dementia before 75 years old irrespective of psychiatric comorbidity (major depression and anxiety disorder). Previous studies showed that age affected associations between mental disorders and dementia [57, 58]. A large population-based case-control study found that the midlife mental disorders were related to dementia with onset at age 65–79, and the association attenuated with age [57]. In another population-based administrative register study of 1.7 million New Zealand citizens observed for 3 decades, people with early-life mental disorders were at elevated risk of subsequent dementia, especially dementia onset in younger age [58]. Possible explanations may be that individuals with mental disorders were more likely to die earlier [59], and thus do not contribute to dementia cases to the oldest age groups. It may also be that older individuals have more opportunity to accumulate risk factors for dementia beyond mental disorders [58]. In addition, the small sample size in the present study leads to low statistical power to find significant association.

The strengths of this study included a representative population, long follow-up from midlife to older age over 50 years, as well as multiple sources of information to detect and diagnose dementia. Some methodological issues need to be considered. *First*, some risk factors might have occurred between baseline and dementia onset. It is likely that stress increases risk for development of other disorders during follow-up, such as cardiovascular factors and depression. That could potentially modify the association between stress-related exhaustion in midlife and dementia incidence. However, these risk factors would most likely decrease the possibility of finding associations in a study with long follow-up as a result of competing risks. *Second*, cumulative attrition is a problem in long-term follow-up studies of dementia. While this problem was, to some extent, alleviated by using medical records and hospital registry data to diagnose dementia in those lost to follow-up, these sources probably underestimate the number of dementia cases. It should be noted, however, that almost all people in Sweden receive their hospital treatment within the public health care system and that the Swedish Hospital Discharge Register covers the entire country. Furthermore, the number of dementia diagnoses detected in the different age groups in our population is what could be expected from other incidence studies [60]. *Finally*, only females were examined in this study. We can therefore not generalize our findings to males or make any statements about potential sex differences regarding the association between stress-related exhaustion and dementia.

## Conclusions

In conclusion, we found that women with stress-related exhaustion in midlife developed dementia at younger age than those without chronic stress in a longitudinal study over 50 years. Women in the former group still presented lower cognitive functions 24 years later even without dementia. Our findings need to be interpreted with caution due to small sample size and should be confirmed in future studies with larger sample size. Our findings also suggest that earlier intervention against stress is important to prevent occurrence of clinical exhaustion, especially for those women who are vulnerable to stress. Individuals who develop stress-related exhaustion need careful long-term follow-up regarding cognitive function.

## Data Availability

The datasets used and/or analysed during the current study are available from the corresponding author on reasonable request.

## Abbreviations

AD: Alzheimer’s disease
CSF: cerebrospinal fluid
GBS-scale: Gottfries-Bråne-Steen scale
ICD: International Statistical Classification of Diseases and Related Health Problems
DSM: Diagnostic and Statistical Manual of Mental Disorders
